# Supplementary figures and images for: Identification of differentially expressed genes involved in amino acid and lipid accumulation of winter turnip rape (Brassica rapa L.) in response to cold stress
Source: PLoS One. 2021 Feb 8;16(2):e0245494. doi: 10.1371/journal.pone.0245494 (PMC7870078; doi:10.1371/journal.pone.0245494)

| 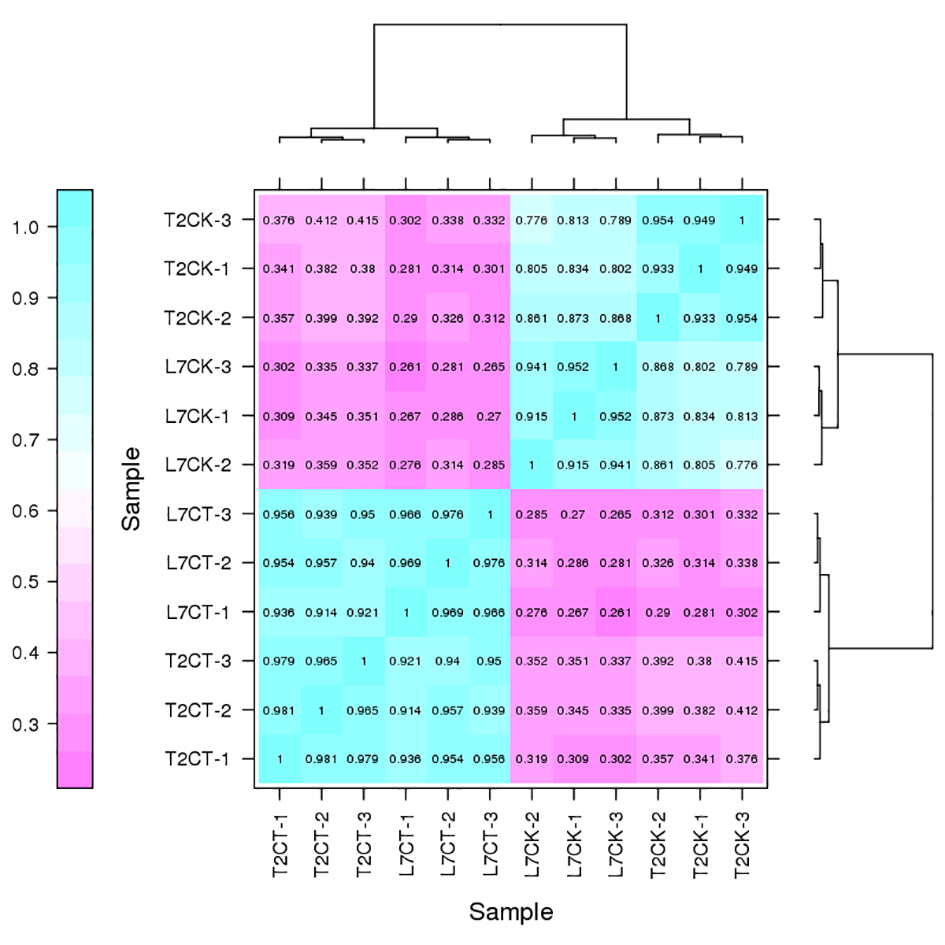 |
| --- |
| **S1 Fig.** The correlations between every two samples among their biological replicates. |

Supplement: S1 Fig — (DOCX) [file pone.0245494.s001.docx]

| 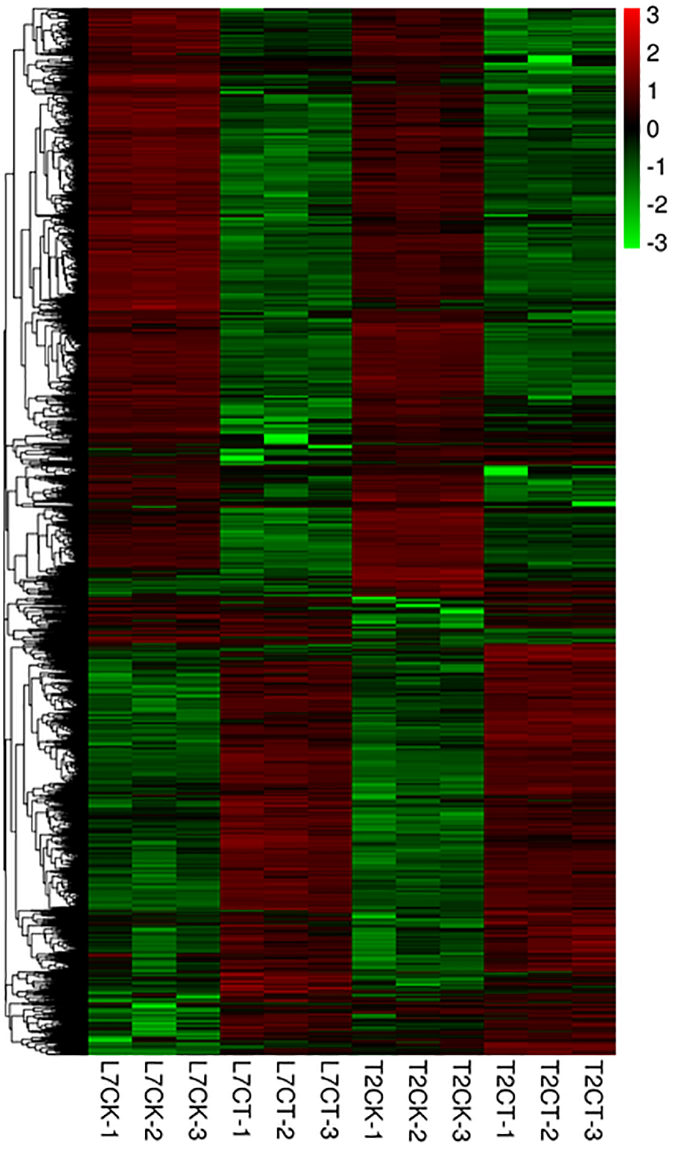 |
| --- |
| **S3 Fig.** Hierarchical cluster map of all differentially expressed genes in two varieties. |

Supplement: S3 Fig — (DOCX) [file pone.0245494.s003.docx]
