# Supplementary material for: Identification of differentially expressed genes involved in amino acid and lipid accumulation of winter turnip rape (Brassica rapa L.) in response to cold stress
Source: PLoS One. 2021 Feb 8;16(2):e0245494. doi: 10.1371/journal.pone.0245494 (PMC7870078; doi:10.1371/journal.pone.0245494)
Supplement: S2 Fig — Grey bars indicate the transcript abundance change based on the FPKM values, according to RNA-seq (left y-axis). Blue lines with standard errors represent the relative expression level, determined by qRT-PCR (right y-axis). The last graph is correlation analysis based on qRT-PCR and RNA-seq data, Pearson’s correlation coefficient is 0.9248 (P < 0.05). (DOCX) [file pone.0245494.s002.docx]

| **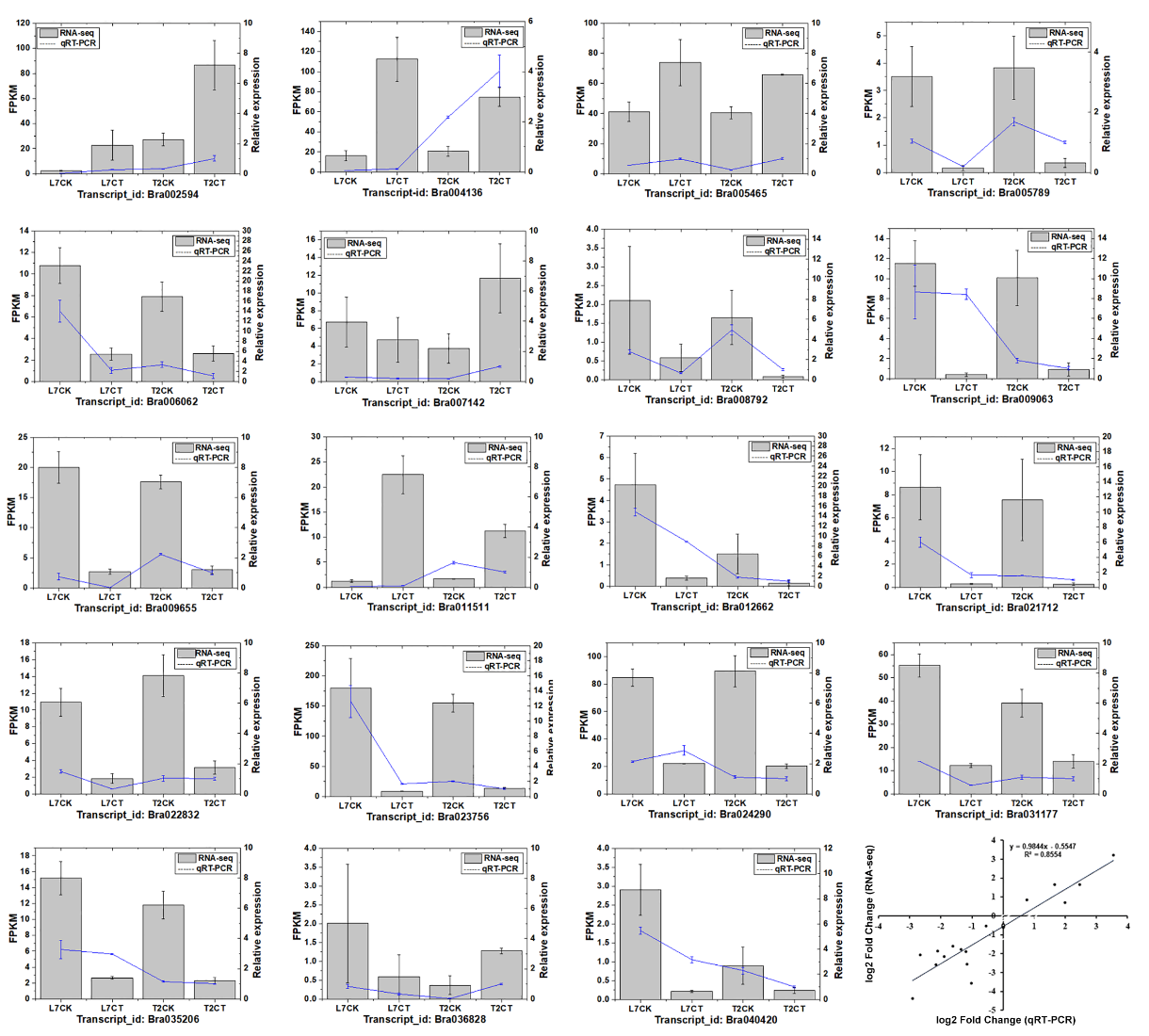** |
| --- |
| **S2 Fig. qRT-PCR validation and correlation analysis of 19 selected genes between the control and cold treatment of two winter turnip rape varieties*.*** Grey bars indicate the transcript abundance change based on the FPKM values, according to RNA-seq (left y-axis). Blue lines with standard errors represent the relative expression level, determined by qRT-PCR (right y-axis). The last graph is correlation analysis based on qRT-PCR and RNA-seq data, Pearson’s correlation coefficient is 0.9248 (*P* < 0.05). |
