# Supplementary material for: Identification of differentially expressed genes involved in amino acid and lipid accumulation of winter turnip rape (Brassica rapa L.) in response to cold stress
Source: PLoS One. 2021 Feb 8;16(2):e0245494. doi: 10.1371/journal.pone.0245494 (PMC7870078; doi:10.1371/journal.pone.0245494)
Supplement: S4 Fig — A. Top 20 enriched pathways for L7 specific cold responsive up-regulated genes; B. Top 20 enriched pathways for L7 specific cold responsive down-regulated genes; C. Top 20 enriched pathways for T2 specific cold responsive up-regulated genes; D. Top 20 enriched pathways for T2 specific cold responsive down-regulated genes. (DOCX) [file pone.0245494.s004.docx]

| 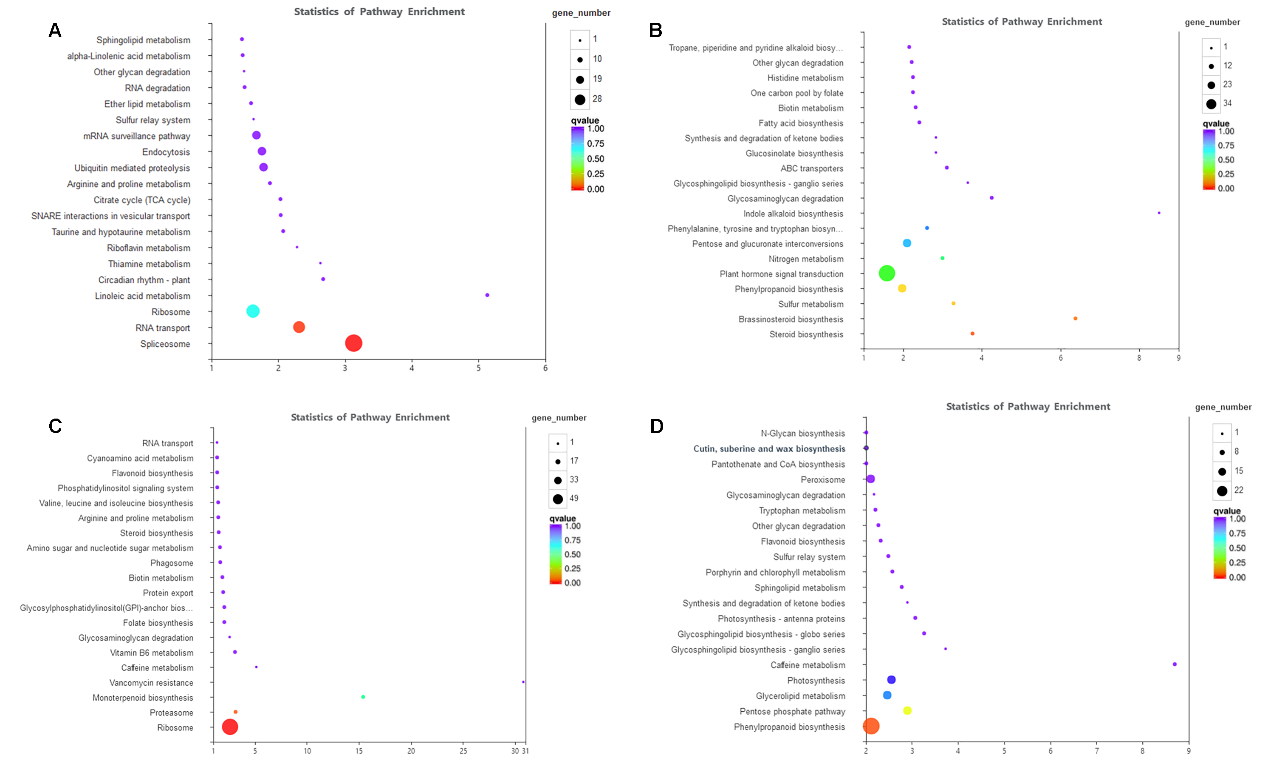 |
| --- |
| **S4 Fig.** Top 20 enriched pathways for cultivar specific differential expressed genes in L7 and T2. A. Top 20 enriched pathways for L7 specific cold responsive up-regulated genes; B. Top 20 enriched pathways for L7 specific cold responsive down-regulated genes; C. Top 20 enriched pathways for T2 specific cold responsive up-regulated genes; D. Top 20 enriched pathways for T2 specific cold responsive down-regulated genes. |
