# Supplementary material for: Identification of differentially expressed genes involved in amino acid and lipid accumulation of winter turnip rape (Brassica rapa L.) in response to cold stress
Source: PLoS One. 2021 Feb 8;16(2):e0245494. doi: 10.1371/journal.pone.0245494 (PMC7870078; doi:10.1371/journal.pone.0245494)
Supplement: S1 Table — (DOCX) [file pone.0245494.s005.docx]

**S1 Table. L7 and T2 cold-resistance capability in ten regions of Gansu province in China**

| Environments | Latitude  (N) | Longitude  (E) | Average temperature of the coldest month (°C) | Mean minimum temperature in coldest month (°C) | L 7 Over-wintering rate | T 2 Over-wintering rate |
| --- | --- | --- | --- | --- | --- | --- |
| Jiuquan | 39°75' | 98°52' | –8.9 | -18.8 | 84 | 44.5 |
| Zhangye | 38.93 | 100.48 | -9.1 | -24.6 | 94.3 | 75.8 |
| Wuwei | 37°93' | 102°64' | –7.2 | -18.2 | 85.85 | 65 |
| Mingqin | 38.62 | 103.08 | -8.10 | -13.3 | 88.71 | 53.28 |
| Jingtai | 37°14' | 104°05' | –6.10 | -17.5 | 88.57 | 58.97 |
| Lanzhou | 36.10 | 103.72 | -4.5 | -15 | 94.0 | 74.67 |
| Huinin | 35°72' | 150°08' | –6.4 | -12.3 | 83.35 | 87.90 |
| Shangchuan | 36°03' | 103°40' | –8.5 | -18 | 81 | 28.50 |
| Huanxian | 36.57 | 107.33 | -7.4 | -13.1 | 84.50 | 46.00 |
| Average | | | | | 87.14 | 59.40 |
