# Supplementary material for: Identification of differentially expressed genes involved in amino acid and lipid accumulation of winter turnip rape (Brassica rapa L.) in response to cold stress
Source: PLoS One. 2021 Feb 8;16(2):e0245494. doi: 10.1371/journal.pone.0245494 (PMC7870078; doi:10.1371/journal.pone.0245494)
Supplement: S2 Table — (DOCX) [file pone.0245494.s006.docx]

**S2 Table. Dilute Original density Standard**

| Concentration | Standard | Dilute and add sample |
| --- | --- | --- |
| 24μmol/L | 5 Standard | 150μl Original density Standard+150μl Standard diluent |
| 12μmol/L | 4 Standard | 150μl 5 Standard+150μl Standard diluent |
| 6μmol/L | 3 Standard | 150μl 4 Standard+150μl Standard diluent |
| 3μmol/L | 2 Standard | 150μl 3 Standard+150μl Standard diluent |
| 1.5μmol/L | 1 Standard | 150μl 2 Standard+150μl Standard diluent |
