# Supplementary material for: Identification of differentially expressed genes involved in amino acid and lipid accumulation of winter turnip rape (Brassica rapa L.) in response to cold stress
Source: PLoS One. 2021 Feb 8;16(2):e0245494. doi: 10.1371/journal.pone.0245494 (PMC7870078; doi:10.1371/journal.pone.0245494)
Supplement: S3 Table — (DOCX) [file pone.0245494.s007.docx]

**S3 Table. Mobile phase gradient elution procedure**

| Time (min) | Flow speed (ml/min) | Mobile phase A (%) | Mobile phase B (%) |
| --- | --- | --- | --- |
| 0.0 | 1.0 | 100 | 0 |
| 14.0 | 1.0 | 85 | 15 |
| 29.0 | 1.0 | 66 | 34 |
| 30.0 | 1.0 | 0 | 100 |
| 37.0 | 1.0 | 0 | 100 |
| 38.0 | 1.0 | 100 | 0 |
| 45.0 | 1.0 | 100 | 0 |
