# Supplementary material for: Identification of differentially expressed genes involved in amino acid and lipid accumulation of winter turnip rape (Brassica rapa L.) in response to cold stress
Source: PLoS One. 2021 Feb 8;16(2):e0245494. doi: 10.1371/journal.pone.0245494 (PMC7870078; doi:10.1371/journal.pone.0245494)
Supplement: S4 Table — (DOCX) [file pone.0245494.s008.docx]

**S4 Table. The primers of 19 transcripts and referenced gene for qRT-PCR validation**

| Gene_ID | Sequence of Primers | Amplification length |
| --- | --- | --- |
| Bra024290 | F: CCGACCAGAAAAGAGGAAT | 114 |
|  | R: AGAACGCAAACGGCATAGT |  |
| Bra011511 | F: TTCTGTGACAACCTTTCCCTC | 107 |
|  | R: CTGGCTCAACCGACAATAA |  |
| Bra004136 | F: AAGTATGCCCAGATTGCT | 112 |
|  | R: TTGGAAACCACATCGTAG |  |
| Bra035206 | F: ATCCGAGTCACCAACAC | 142 |
|  | R: GCTGAAAATCGCCATAC |  |
| Bra009655 | F: ATGGGAGGGTTCGTTCT | 187 |
|  | R: CCGCTTGGCTACTGTTC |  |
| Bra012662 | F: ACAACACTGGTCCCTAA | 149 |
|  | R: ACATACTTCACTTTTCCTA |  |
| Bra006062 | F: AAGTCAAAGTCCTCCTAC | 167 |
|  | R: GACCAAACCTACTAATCC |  |
| Bra031177 | F: AGTTGGAACCACCGACAG | 117 |
|  | R: CGGACCAGACCCATAGAA |  |
| Bra005789 | F: AACGGGGAGGATACTGGGGATA | 197 |
|  | R: AAGGCGGATGATACGGCAC |  |
| Bra008792 | F: TACTGTCCTCCGTCTCG | 119 |
|  | R: AGTCAAGGTGGGTGTCA |  |
| Bra007142 | F: CCTGGTGCTTCCATCCTC | 124 |
|  | R: CTGCCTCTGCCAACAACT |  |
| Bra036828 | F: CGATGTTGGTGGGAAAAG | 153 |
|  | R: TCAGGAGGTAAGGCGAAG |  |
| Bra005465 | F: CTCTGGTGATGCTGTGC | 195 |
|  | R: GCCTTGGGGTTGTAGTA |  |
| Bra040420 | F: TCGTCCCCGTTCTCATCT | 178 |
|  | R: TTCCACCCATCGTCGTAA |  |
| Bra002594 | F: GAGAAGGCGGAGAATGG | 126 |
|  | R: TTGGCTAACTCGTAAATCA |  |
| Bra023756 | F: TCGGATTTCAAAGGAGG | 161 |
|  | R: ACCGCTGCGTGAGACAA |  |
| Bra021712 | F: GAAGAAACCCCAAACGC | 167 |
|  | R: AGCCAAACCCTAATCCC |  |
| Bra022832 | F: AGAAACCCAAAACGACGAC | 157 |
|  | R: GAGCCAAACCCTAATCCC |  |
| Bra009063 | F: GAACAGGCAGATAAGCG | 110 |
|  | R: TTCGGAAGAGTTGAAAGTA |  |
| Actin-7 | F: TGTGCCAATCTACGAGGGTTT | 137 |
|  | R: TTTCCCGCTCTGCTGTTGT |  |
