# Supplementary material for: Identification of differentially expressed genes involved in amino acid and lipid accumulation of winter turnip rape (Brassica rapa L.) in response to cold stress
Source: PLoS One. 2021 Feb 8;16(2):e0245494. doi: 10.1371/journal.pone.0245494 (PMC7870078; doi:10.1371/journal.pone.0245494)
Supplement: S5 Table — (DOCX) [file pone.0245494.s009.docx]

**S5 Table. The annotation information of 19 transcripts for qRT-PCR**

| **Gene_ID** | **COG_class_annotation** | **KEGG_pathway_annotation** | **NR_annotation** |
| --- | --- | --- | --- |
| **Bra024290** | General function prediction only | Pyrimidine metabolism (ko00240); beta-Alanine metabolism (ko00410); Pantothenate and CoA biosynthesis (ko00770) | beta-ureidopropionase [Arabidopsis thaliana] |
| **Bra011511*** | Energy production and conversion | Glycolysis / Gluconeogenesis (ko00010); Pentose and glucuronate interconversions (ko00040); Ascorbate and aldarate metabolism (ko00053); Fatty acid degradation (ko00071); Valine, leucine and isoleucine degradation (ko00280); Lysine degradation (ko00310); Arginine and proline metabolism (ko00330); Histidine metabolism (ko00340); Tryptophan metabolism (ko00380); beta-Alanine metabolism (ko00410); Glycerolipid metabolism (ko00561); Pyruvate metabolism (ko00620); Limonene and pinene degradation (ko00903) | putative aldehyde dehydrogenase [Brassica rapa] |
| **Bra004136*** | Amino acid transport and metabolism | Alanine, aspartate and glutamate metabolism (ko00250); beta-Alanine metabolism (ko00410); Taurine and hypotaurine metabolism (ko00430); Butanoate metabolism (ko00650) | glutamate decarboxylase 2 [Brassica juncea] |
| **Bra035206** | Energy production and conversion; Coenzyme transport and metabolism; General function prediction only | Glycine, serine and threonine metabolism (ko00260); Glyoxylate and dicarboxylate metabolism (ko00630) | oxidoreductase family protein [Arabidopsis lyrata subsp. lyrata] |
| **Bra009655** | Amino acid transport and metabolism | Glycine, serine and threonine metabolism (ko00260); Biosynthesis of amino acids (ko01230) | homoserine kinase [Arabidopsis lyrata subsp. lyrata] |
| **Bra012662** | General function prediction only | Fatty acid elongation (ko00062); Fatty acid metabolism (ko01212) | palmitoyl protein thioesterase family protein [Brassica oleracea] |
| **Bra006062** | -- | Fatty acid elongation (ko00062); Biosynthesis of unsaturated fatty acids (ko01040); Fatty acid metabolism (ko01212) | PASTICCINO 2 [Brassica rapa subsp. pekinensis] |
| **Bra031177** | General function prediction only | Pentose and glucuronate interconversions (ko00040); Fructose and mannose metabolism (ko00051); Galactose metabolism (ko00052); Glycerolipid metabolism (ko00561) | hypothetical protein ARALYDRAFT_480985 [Arabidopsis lyrata subsp. lyrata] |
| **Bra005789** | General function prediction only | Steroid biosynthesis (ko00100); Glycerolipid metabolism (ko00561); Glycerophospholipid metabolism (ko00564); Ether lipid metabolism (ko00565); Arachidonic acid metabolism (ko00590); Linoleic acid metabolism (ko00591); alpha-Linolenic acid metabolism (ko00592) | sugar-dependent1 [Arabidopsis lyrata subsp. lyrata] |
| **Bra008792*** | -- | Flavonoid biosynthesis (ko00941); Circadian rhythm - plant (ko04712) | chalcone synthase [Brassica rapa var. parachinensis] |
| **Bra007142*** | -- | Flavonoid biosynthesis (ko00941) | chalcone-flavanone isomerase 1 protein [Brassica rapa subsp. oleifera] |
| **Bra036828*** | General function prediction only | Flavonoid biosynthesis (ko00941) | flavanone 3-hydroxylase 1 protein [Brassica rapa subsp. oleifera] |
| **Bra005465** | -- | Plant hormone signal transduction (ko04075) | auxin response factor 3-1 [Brassica rapa subsp. pekinensis] |
| **Bra040420** | Lipid transport and metabolism | Plant hormone signal transduction (ko04075) | gibberellin receptor 1B [Brassica napus] |
| **Bra002594** | Signal transduction mechanisms | Plant hormone signal transduction (ko04075) | putative protein phosphatase 2C 78 [Arabidopsis thaliana] |
| **Bra023756** | Signal transduction mechanisms | Plant hormone signal transduction (ko04075) | ethylene receptor [Brassica oleracea] |
| **Bra021712** | -- | Plant hormone signal transduction (ko04075) | hypothetical protein ARALYDRAFT_482050 [Arabidopsis lyrata subsp. lyrata] |
| **Bra022832** | -- | Plant hormone signal transduction (ko04075) | ethylene reponse factor-like AP2 domain transcription factor [Arabidopsis thaliana] |
| **Bra009063** | -- | Plant hormone signal transduction (ko04075) | 80A08_2 [Brassica rapa subsp. pekinensis] |

Note: The GeneID is marked with an asterisk to indicate that they have already been reported to be related with cold stress.
